# Allergenicity of partially hydrolyzed whey and casein formulas evaluated by ImmunoCAP inhibition assay and basophil activation test

**DOI:** 10.3389/falgy.2023.1207924

**Published:** 2023-07-21

**Authors:** Takeshi Matsubara, Fuka Ishikawa, Chisato Inuo, Mayumi Fujita, Ayumi Tsukahara, Takahiro Koyama, Hiroshi Iwamoto, Kazuhiro Miyaji

**Affiliations:** ^1^ Health Care & Nutrition Science Institute, R&D Section, Morinaga Milk Industry Co., Ltd., Zama, Kanagawa, Japan; ^2^ Department of Allergy, Kanagawa Children’s Medical Center, Yokohama, Kanagawa, Japan

**Keywords:** partially hydrolyzed formula, immunoCAP inhibition assay, basophil activation test, cow’s milk allergy, allergenicity

## Abstract

**Background:**

When exclusive breastfeeding is not possible, partially hydrolyzed formula (PHF) is often used as a starter formula for infants. Some children develop allergic symptoms, including anaphylaxis, after the first intake of cow protein. Therefore, the tolerability of PHF in infants with cow's milk allergy (CMA) is important information. Partially hydrolyzed whey formula (PHWF) is well characterized, but those containing both whey and casein are also available. We evaluated the characteristics of two whey and casein PHFs, PHF1 and PHF2, *in vitro* and *ex vivo*, and compared them with a PHWF, PHWF1.

**Methods:**

Residual antigenicity of β-lactoglobulin (β-LG) and casein in the formulas was measured using ELISA. The molecular weight profile was determined using high-pressure liquid chromatography. IgE reactivity and allergenic activity of the formulas were evaluated by ImmunoCAP inhibition assay and by basophil activation test using blood from patients with CMA, respectively.

**Results:**

All the participants (*n* = 10) had casein-specific IgE. The antigenicity of β-LG in PHF1 was similar to that in PHWF1, but it was slightly higher than that in PHWF1 for casein. PHF1 had a higher IgE reactivity than PHWF1. However, PHF1 and PHWF1 had a similar ability to activate basophils. PHF2 had lower antigenicity of casein and β-LG, IgE reactivity and basophil activation than PHWF1.

**Conclusion:**

These results suggest that the tolerability of PHF1 and PHF2 in patients with CMA is similar to and higher than that of PHWF1, respectively, and that the degree of IgE binding to PHFs does not necessarily correspond to basophil activation.

## 1. Introduction

Although the causative foods for food allergies vary by age and country, cow's milk is the major causative food in children in many countries (1). Cow's milk is the source of primary ingredients in infant formula and serves as an important source of nutrition for infants who cannot ingest a sufficient amount of breast milk.

Cow's milk-based infant formula is classified into conventional cow's milk formula (CMF), partially hydrolyzed formula (PHF), and extensively hydrolyzed formula (EHF) according to the degree of hydrolysis of milk proteins. PHF was designed and developed to reduce the sensitizing capacity of cow milk proteins while maintaining the palatability of the formula at a reduced processing cost compared to EHF (2). Most of the peptides in PHF are 3,000–10,000 Da in molecular size (3), and the antigenicity of PHF measured by β-lactoglobulin (β-LG)-specific sandwich ELISA is approximately 10–300 times lesser than that of CMF, based on the results of previous reports (2, 4).

Multiple clinical trials have shown the reducing effect of PHF on atopic dermatitis compared to that of CMF (5, 6), although systematic reviews have indicated insufficient evidence of this effect (7, 8). In contrast, no study clearly showed the preventive tendency or effect of PHF on cow's milk allergy (CMA) due to not matching to strict criteria for diagnosing IgE-mediated CMA (8, 9).

Although not as well as EHF, the reactivity of PHF in children with CMA is substantially reduced. It is reported that approximately 40%–75% of children with CMA can consume PHF (10–12), and the symptoms are mild compared to CMF when children develop allergic symptoms after ingesting PHF (13). Additionally, it has been reported that 7%–15% of infants with CMA developed symptoms at the first intake of cow's milk protein (14) and that the most infants with CMA anaphylaxis was caused by the first intake of formula after breastfeeding discontinuation (15). Furthermore, about half of the infants who suffer from allergies are not infants born to families with atopic disease (16), and none of the data suggest that PHF may be potentially harmful to healthy infants (17). From these points, PHF is considered to be suitable as a starter formula regardless of allergy risk status when the presence or absence of CMA in an infant is unknown.

For the above reasons, the tolerability of starter formulas in infants with CMA is important information. Milk proteins are broadly divided into casein and whey proteins. Although both contain some milk allergens, most children with CMA have higher specific IgE values for casein than for whey proteins (18, 19). Partially hydrolyzed whey formula (PHWF) is predominant in the global market for PHF, but those containing both whey and casein are also available. It has been believed that a considerable proportion of patients with CMA could tolerate PHF based on some reports using a specific PHWF (10, 11, 20). In contrast, the tolerability of PHF containing both casein and whey proteins remains to be clarified. Notably, very few reports have compared various products with the same patient background.

Specific IgE values to food allergens indicate the individual states of sensitization to the allergen, and the IgE levels do not always correspond to clinical symptoms (19). The double-blinded placebo-controlled food challenge (DBPCFC) test is the gold standard for determining whether food allergens can be ingested. However, the DBPCFC test has the potential risk of anaphylaxis and is time-consuming and expensive. In the past two decades, the basophil activation test (BAT) has attracted attention as an *ex vivo* testing method that is highly correlated with the clinical symptoms of food allergy (21). In fact, BAT is considered reliable for diagnosing IgE-mediated CMA and can be an alternative to DBPCFC (22). Therefore, it is speculated that the degree of IgE-reactivity to allergen and basophil activation by the allergen do not necessarily correspond.

In this study, we aimed to estimate the tolerability of PHF containing both casein and whey protein hydrolysates in children with CMA. To achieve this, two PHFs containing both hydrolysates were evaluated by CAP inhibition assay and BAT using blood from the children and compared with a PHWF. The tolerability data of the two PHFs evaluated relative to PHWF, which has a wealth of knowledge, will be very useful for the safe use of them as starter formulas.

## 2. Materials and methods

### 2.1. Study design and participants

This was a cross-sectional study of patients with CMA evaluating basophil activation and IgE reactivity to milk proteins in infant formulas using peripheral blood samples. Participants were recruited from outpatients at Kanagawa Children's Medical Center. The inclusion criteria were as follows: (I) aged 2–15 years; (II) history of immediate symptoms to milk components; and (III) milk-specific antibodies (>3.5 U_A_/ml). The exclusion criteria were as follows: (I) taking antihistamines within 3 days before the scheduled blood sampling date and (II) taking drugs, such as steroids, that act on the immune system within 1 week before the date. Since the aim of this study was to evaluate the allergenicity of formulas using the blood of children with CMA, the age range was set to 2–15 years old. This study was conducted between January and March 2022 and was registered in the UMIN Clinical Trials Registry (study ID: UMIN000046543).

### 2.2. Ethics and informed consent

The study procedures were explained to all participants and their parents, and written informed consent was obtained at enrollment. This study was conducted in accordance with the Declaration of Helsinki and approved by the ethics review board of Kanagawa Children's Medical Center (No. 132-2).

### 2.3. Milk formulas

Four powdered milk formulas were used: PHF1 (PHP; PT Kalbe Morinaga Indonesia), PHF2 (E-akachan; Morinaga Milk Industry Co., Ltd., Japan), PHWF1 (NAN pH Pro-1; PT Nestle Indonesia), and CMF1 (Hagukumi; Morinaga Milk Industry Co., Ltd., Japan).

### 2.4. ELISA

The residual antigenicity of casein (CN) and β-lactoglobulin (β-LG) in each formula was determined using commercial ELISA kits (FASPEK II, Morinaga Institute of Biological Science, Japan) according to the manufacturer's instructions. The ELISA kits are based on polyclonal antibodies.

### 2.5. High-pressure liquid chromatography (HPLC)

Formulas were diluted with 0.1 M phosphate buffer (pH 7.4) and centrifuged (2,000×*g*, 25°C, 15 min) followed by collecting the middle layer as defatted fractions. Subsequently, they were filtrated with a 0.22 µm filter (Millex-GV, Merck Life Science, Germany) and applied to HPLC (LC-20AD, Shimadzu, Japan) with a poly-hydroxyethyl aspartamide column (PolyLC, Columbia, MD, USA). The molecular weight pattern of the formulae was determined as previously described (23). Immunoglobulin G, lactoperoxidase, ovalbumin, chymotrypsinogen A, ribonuclease A, bovine insulin, basitracin, oxytocin, D-Ala, Met-enkephalinamide, L-methionine, and L-glutamine were used for molecular weight calibration.

### 2.6. Blood sampling

At the time of blood collection from the participants to measure cow's milk allergen-specific IgE for usual care, additional amounts (7 ml) of blood samples were also collected for this study. For collection, 3 and 4 ml of the samples were collected for heparinized blood samples to be used in the BAT and for serum samples to be used in the ImmunoCAP inhibition assay, respectively.

### 2.7. Measurement of serum cow's milk allergen-specific IgE

Serum levels of IgE antibodies to milk (f2), casein (f78), β-LG (f77), and α-lactalbumin (f76) were measured using ImmunoCAP (Thermo Fisher Scientific, Sweden), according to the manufacturer's instructions.

### 2.8. ImmunoCAP inhibition assay

Formulas used as inhibitors were diluted with 0.1 M phosphate buffer (pH 7.4). Serum samples were diluted with IgE Sample Diluent (Thermo Fisher Diagnostics, Tokyo, Japan) to make cow's milk-specific IgE 4–10 U_A_/ml. The diluted formulas and serum samples were mixed in a 3:2 ratio and incubated at room temperature for 2 h, as previously reported (24). Incubation of serum samples with the diluent served as a negative control. IgE reactivity to cow milk (f2) was measured using the ImmunoCAP system with Phadia 200 (Thermo Fisher Diagnostics, Tokyo, Japan). The extent of inhibition was calculated using the following formula: % inhibition = 100−[anti-milk IgE of serum sample incubated with inhibitor (U_A_/ml)] × 100/anti-milk IgE of serum sample incubated without inhibitor (U_A_/ml). The half-maximal inhibitory concentration (IC_50_) values were calculated using statistical software (JMP version 13.0; SAS Institute).

### 2.9. Basophil activation test (BAT)

BAT was performed at BML Inc. (Saitama, Japan) using an allergenicity kit (Beckman Coulter, CA, USA) according to the manufacturer's instructions. Briefly, after incubating heparinized whole blood with the antigen at 37°C for 15 min, basophil activation was assessed using antibodies against CD3, chemoattractant receptor-homologous molecule on Th2 cells (CRTH2), and CD203c. PBS and anti-IgE antibodies were used as the negative and positive controls, respectively. Serially diluted formula samples were used to assess basophil activation for formulas. CD203c expression in at least 300 basophils (CD3^−^CRTH2^+^ cells with FSC/SSC characteristics of lymphocytes) was analyzed using flow cytometry. Basophil activation was expressed as the net percentage of CD203c positive basophils above the threshold defined by the negative control. Basophils were defined as non-responders when the percentage of basophils activated by anti-IgE was less than 10% (13). The area under the curve (AUC) of the activated basophils with a log-formula concentration axis was calculated for each participant.

### 2.10. Statistical analysis

Data are expressed as mean ± standard deviation (SD). The difference compared to PHWF1 was analyzed by paired *t*-test using Excel with the Holm-Benferroni method for correction of multiple comparisons. The correlation between specific IgE values and AUC of the activated basophils was analyzed using the Pearson correlation analysis using JMP version 13 (SAS Institute, Cary, NC, USA). Statistical significance was set at *P* < 0.05.

## 3. Results

### 3.1. Residual antigenicity and molecular weight profile of formulas

The residual antigenicity of β-LG and casein was determined using commercially available ELISA kits (Table 1). The antigenicity of β-LG and casein in the three PHFs was 30-to-150- and 460-to-6,700-fold lesser than that in CMF1, respectively. Compared to PHWF1, PHF1 had 5-fold higher casein antigenicity and almost the same β-LG level. PHF2 had 3- and 5-fold lesser antigenicity for casein and β-LG than PHWF1, respectively.

**Table 1:** Characteristics of formulas.

Name	Residual antigenicity in ready-to-use product [µg/ml]^a^		Molecular weight (Da)^b^					
	β-LG	Casein	<200	200–1,000	1,000–3,500	3,500–5,000	5,000–10,000	10,000<
PHF1	75.4	15.6	19.8	60.6	19.1	0.99	0.46	0.14
PHF2	16.9	1.08	21.9	59.4	16.7	1.24	1.08	1.12
PHWF1	82.5	3.14	7.16	66.8	23.1	1.02	1.04	1.89
CMF1	2.60 × 10^3^	7.28 × 10^3^	11.0	14.5	13.8	2.81	5.53	56.2

^a^ Residual antigenicity of β-LG and casein in formulas was measured by ELISA.

^b^ Molecular weight profile of formulas was determined by high-pressure liquid chromatography. Data are expressed as the percentage of total protein or peptide.

The molecular weight profiles of the formulae were determined using HPLC (Table 1). In CMF1, 56% of the proteins were >10,000 Da. In the PHFs, more than 97% of the proteins were below 5,000 Da. Compared to PHWF1, PHF1 had lower proportions of proteins with molecular masses of 5,000–10,000 Da and above 10,000 Da. PHF2 also had a lower percentage of proteins with molecular weights above 10,000 Da.

### 3.2. Participants

Ten children were enrolled, including seven boys and three girls, with an average age of 8.24 ± 2.70 years. Sixty percent of them suffered from food allergies other than cow's milk. Specifically, eggs (60%), wheat (20%), buckwheat (10%), and sesame (10%). They had specific IgE to milk (40.0 ± 28.3 U_A_/ml), casein (84.3 ± 179 U_A_/ml), β-LG (11.3 ± 16.4 U_A_/ml), and α-LA (5.66 ± 8.55 U_A_/ml) (Table 2). Cases were missing in the ImmunoCAP inhibition assay (Patient 7) and BAT (Patient 8) because of insufficient blood volume.

**Table 2:** Characteristics of participants with cow's milk allergy.

Patient ID	Age (years, months)	Sex	Food allergy other than to CM	Specific IgE (U_A_/ml)			
				CM	casein	β-LG	α-LA
1	6 y3 m	M	–	50.0	44.5	6.18	9.08
2	9 y2 m	M	Egg, wheat	31.1	28.0	NA^a^	NA^a^
3	3 y10 m	M	–	16.8	19.9	0.69	0.24
4	5 y0 m	M	–	11.7	5.08	8.51	4.64
5	11 y8 m	M	Egg	14.8	14.8	0.80	1.38
6	8 y9 m	F	Egg, wheat	14.8	17.3	0.08	0.31
7	11 y7 m	M	–	94.4	589	26.9	50.5
8	8 y5 m	F	Egg	10.2	8.21	0.1	0.26
9	7 y0 m	M	Egg, Buckwheat	50.2	41.2	5.81	19.5
10	10 y9 m	F	Egg, sesame	65.6	74.6	1.84	16.1

^a^ Not applied.

### 3.3. Reactivity of cow's milk-specific IgE to formulas

**Figure 1:**
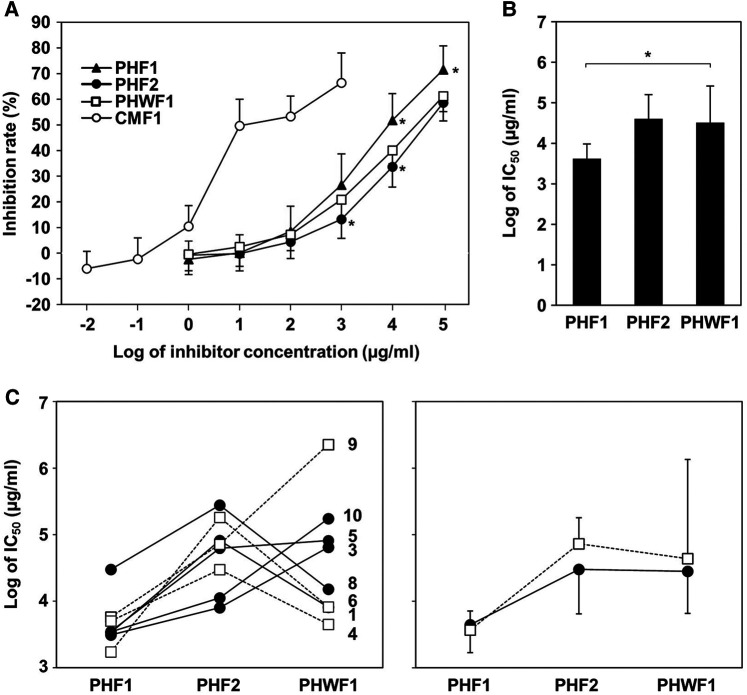
ImmunoCAP inhibition of the formulas for milk protein using sera from the participants with cow's milk allergy. Serially diluted formulas were mixed with the serum samples from participants with cow's milk allergy and incubated. Milk-specific IgE was measured using the ImmunoCAP system. (**A**) Inhibition rate of milk-specific IgE by diluted formula samples. (**B**) The log values of half-maximal inhibitory concentration (IC_50_) of PHFs. Data are represented as the mean ± SD (*n* = 9). The asterisks indicate significant differences from PHWF1. (**C**) Left panel: The log IC_50_ of PHFs in individual patients. Filled circles with a solid line; low β-LG subgroup (β-LG-specific IgE <3.5 U_A_/ml, Patients 3, 5, 6, 8, and 10). Blank squares with a dashed line; high β-LG subgroup (β-LG-specific IgE ≥3.5 U_A_/ml, Patients 1, 4, and 9). Right panel: Mean ± SD of log IC_50_ of PHFs in each subgroup.

To evaluate the reactivity of cow milk-specific IgE to the formulas, an inhibition assay was performed using the CAP system. CMF1 inhibited the binding of IgE to milk antigen (f2) at low concentrations. Less inhibition was observed in PHF1 at 10^4^ and 10^5^ µg/ml and in PHF2 at 10^3^ and 10^4^ µg/ml compared to PHWF1 (Figure 1A). The IC_50_ values for CMF1, PHF1, PHF2, and PHWF1 were 2.62, 4.16 × 10^3^, 4.04 × 10^4^, and 3.25 × 10^4^ μg/ml, respectively. The log IC_50_ value of PHF1 was lower than that of PHWF1 (Figure 1B). These results indicated higher IgE reactivity to PHF1 and the same or lower tendency of reactivity to PHF2 than to PHWF1. Patients were divided into two subgroups, high β-LG (β-LG-specific IgE ≥3.5 U_A_/ml) and low β-LG (β-LG-specific IgE <3.5 U_A_/ml). The log IC_50_ values of PHF1 in Patients 1 and 4 of high β-LG and Patients 6 and 8 of low β-LG were almost the same as those of PHWF1. No difference was observed in the pattern of log IC_50_ values of three formulas between the two subgroups (Figure 1C).

### 3.4. Basophil responsiveness to stimulation with formulas

**Figure 2:**
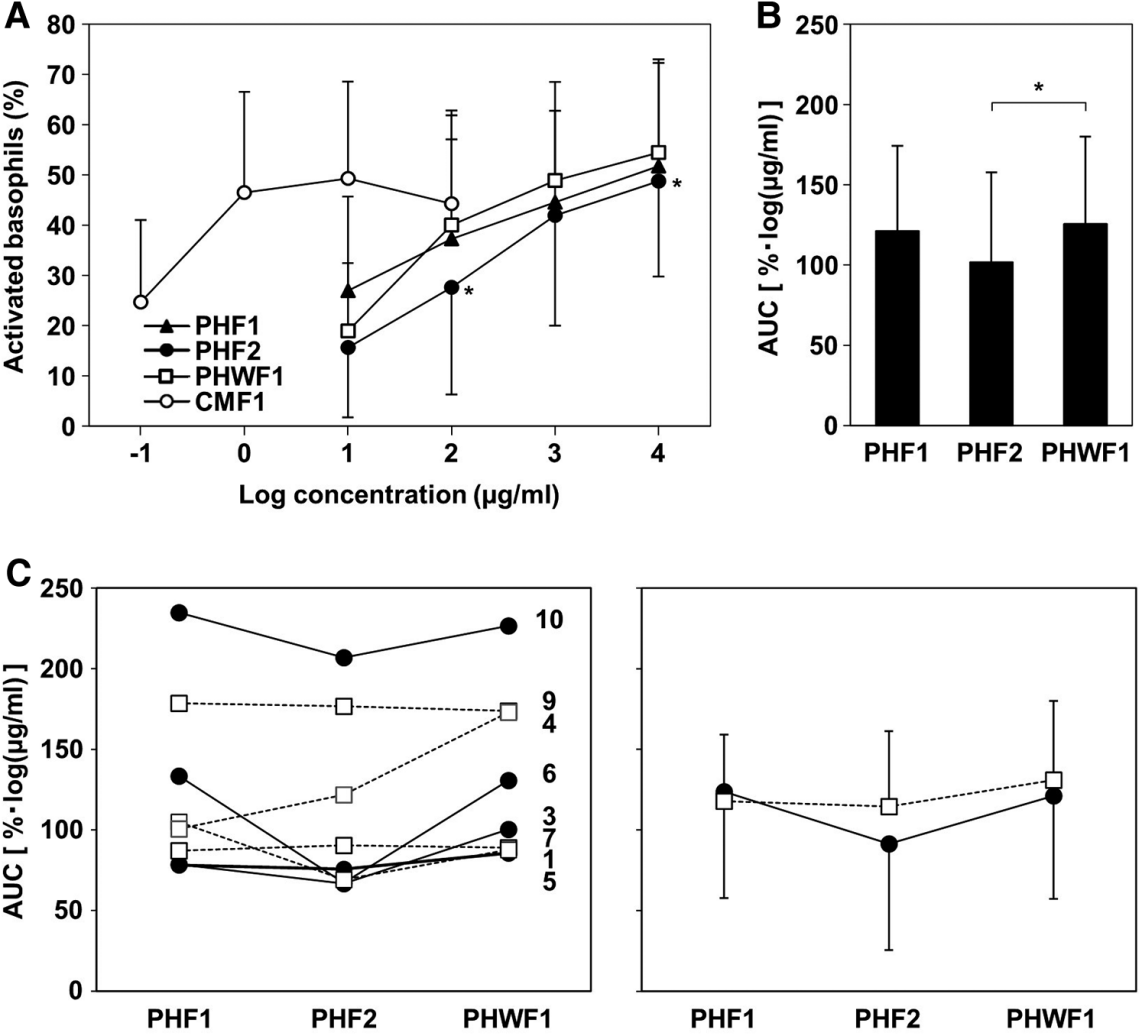
Basophil activation in response to the formulas. The basophil activation test was performed using a serially diluted formula. (**A**) The percentage of CD203c upregulated basophils. (**B**) The area under the curve (AUC) of activated basophils with a log formula concentration axis. Data are represented as the mean ± SD (*n* = 9). The asterisks indicate significant differences from PHWF1. (**C**) Left panel: The AUC of basophils activated by PHFs in individual patients. Filled circles with solid line; low β-LG subgroup (β-LG-specific IgE <3.5 U_A_/ml, Patients 3, 5, 6, and 10). Blank squares with a dashed line; high β-LG subgroup (β-LG-specific IgE ≥3.5 U_A_/ml, Patients 1, 4, 7, and 9). Right panel: Mean ± SD of the AUC of activated basophils in each subgroup.

To evaluate the ability of the formulas to induce activation of basophils from patients with CMA, BAT was performed using their blood. None of the participants had non-responder basophils. All formulas activated basophils in a concentration-dependent manner. CMF1 activated basophils at low concentrations. PHF2 was less effective than PHWF1 at activating basophils at concentrations of 10^2^ and 10^4^ µg/ml. PHF1 and PHWF1 showed no differences at any concentration tested (Figure 2A). The AUC of the activated basophils was significantly lower when stimulated with PHF2 than when stimulated with PHWF1. No differences were observed between the AUCs of PHF1 and PHWF1 (Figure 2B). There was no significant correlation between specific IgE to milk, casein, β-LG, or α-LA and AUC of each PHF. The AUC of PHF2 was the smallest among the 3 PHFs in all the patients of the low β-LG subgroup, while 3 (Patients 4, 7, and 9) of 4 patients in the high β-LG subgroup did not show the tendency (Figure 2C, left panel). Reflecting these results, the average AUC of PHF2 in the low β-LG subgroup was the smallest between the PHFs, and those of the 3 PHFs in the high β-LG subgroup were almost the same (Figure 2C, right panel).

## 4. Discussion

Because a starter formula is used when the presence of absence of CMA in an infant is unknown, tolerability of the formula in infants with CMA is important information. Regarding the allergenicity of PHF, there are some reports on PHWF, but there are few reports on the use of casein and whey protein hydrolysates. In this study, we compared the allergenicity of two PHFs containing casein and whey proteins, PHF1 and PHF2, with PHWF1, a typical PHWF product, using *in vitro* and *ex vivo* methods. To the best of our knowledge, this is the first report evaluating the allergenicity of PHF using both the ImmunoCAP inhibition assay and basophil activation test using blood from patients with CMA.

PHF2 had lower antigenicity of casein and β-LG by sandwich ELISA and lower allergenicity evaluated by IgE binding and BATs than PHWF1. This suggests that PHF2 may be less reactive than PHWF1 when ingested by children with CMA. Clinical studies evaluating the tolerability of PHF by an oral challenge test in patients with CMA have been reported, and a PHWF from the same manufacturer as PHWF1 used in this study and PHF2 can be ingested by 40%–60% (10, 11, 20) and 75% (12) of patients, respectively. It is difficult to compare the tolerability of both formulas from these reports because of the lack of comparison in the same participants, the limited number of cases, the different countries where they were performed, and the possible differences in the severity of CMA. However, the fact that a higher percentage of patients tolerated PHF2 is considered to follow the same trend as the results of this study.

The participants in this study showed many individual differences in specific IgE to milk components. When they were divided into two subgroups based on β-LG-specific IgE values, the trends on basophil activation by the PHFs but on IgE binding to them differed between the groups. This result suggests that different IgE profiles on milk components may have different tendencies toward basophil activation. Further studies with more number of patients to confirm this possibility and elucidate the mechanism are required.

PHF1 was similar in the antigenicity of β-LG determined by sandwich ELISA compared to that of PHWF1, but slightly higher than that of casein. In addition, all participants in this study had casein-specific IgE, and PHF1 inhibited the binding of IgE to milk antigens more than PHWF1 did. These results indicate that many IgE epitopes of casein remain in PHF1. In contrast, PHF1 and PHWF1 had similar abilities to activate basophils, although the participants were more likely to develop basophil activation to casein. Although casein is not used as a raw material for PHWF, casein with an intact size was reportedly detected in PHWF (25). In addition, casein was reported to be less hydrolyzed than whey proteins and remained intact in whey protein hydrolysates even after prolonged hydrolysis, and the hydrolysates activated basophils from patients with high casein-specific IgE (26). These results suggest that whey protein hydrolysates used as media for PHWFs contain casein components that induce casein-specific basophil activation. In contrast, comparing the molecular weight distribution of the protein hydrolysates contained in PHF1 and PHWF1, PHF1 had fewer peptides with a molecular size of 5,000 Da or larger, especially 10,000 Da or larger, than PHWF1. Therefore, the similar ability of both formulas to activate basophils, regardless of the different number of casein IgE epitopes, may be due to the difference in the molecular weight of casein in the formulas, that is, the difference in the number of multivalent epitopes causing basophil degranulation.

This study compared the allergenicity of PHF1 and PHF2 with that of PHWF1 by ImmunoCAP inhibition assay and BAT using blood from children with CMA. This study has two strengths. First, we performed BAT, which is highly correlated with clinical symptoms (21, 22), using blood from CMA patients and compared the PHFs containing both casein and whey protein hydrolysates with PHWF, which provides a wealth of knowledge. Although the previous study compared basophil activation between some PHWFs in an RBL-2H3 cell line passively sensitized with animal IgE (27), this is the first report comparing PHFs using basophils from CMA patients. Second, it could be said that it is novel to compare the allergenicity of multiple PHFs in the same patient with CMA by evaluating the relationship between the two assays. Besides the tolerability of PHF1 and PHF2, this study suggests that the degree of IgE binding to PHFs does not necessarily correspond to that of basophil activation and that the specific IgE profile may be related to the reactivity to PHFs. However, this study had several limitations. First, we evaluated immunological properties using only *in vitro* and *ex vivo* experiments. Although BAT shows a high correlation with the oral challenge test (21, 28), results obtained by the oral challenge test using the PHFs may not always be similar to those of the BAT in this study. Therefore, an *in vivo* evaluation, such as an oral challenge, is required. Second, some patients with CMA are reported to be sensitized only to whey proteins (25), although all participants in this study were sensitized to casein. In addition, owing to the small number of patients, the results of this study may not be applicable to all patients with CMA. Third, based on the antigenicity of β-LG and casein determined by sandwich ELISA, it is speculated that the ability of PHF1 and PHF2 to activate basophils is mainly due to the whey protein hydrolysate. However, it is unclear whether this speculation is true because each hydrolysate (whey protein hydrolysate or casein hydrolysate) was not analyzed separately. Finally, a comparison between the products was performed using only one lot for each product. Product antigenicity may vary, to some extent, between lots (27, 29). To improve accuracy, it is preferable to compare multiple product lots.

In this study, although PHF1 had more IgE epitopes of cow's milk proteins than PHWF1, the ability of PHF1 to activate basophils from patients with CMA was similar to that of PHWF1. This suggests that the risks of developing allergies when ingesting PHF1 or PHWF1 are similar. PHF2, which was previously reported to be effective in oral immunotherapy (30), was shown to have lower IgE epitopes and basophil-activating ability than PHWF1. Therefore, PHF1 and PHF2 are considered to be highly safe starter formulas. Recent studies on the effects of the early introduction of cow's milk on CMA suggests that supplementation with CMF in the first week of life, followed by a period of avoidance due to exclusive breastfeeding, is associated with an increased risk of CMA (31). Therefore, PHF intake during the first week might reduce sensitization to cow's milk proteins.

Food allergies are partially attributed to the loss or lack of tolerance to food allergens (32). Hydrolyzed formulas for treating or preventing CMA require low allergenicity and the ability to activate T cells to induce oral tolerance (33). Some hydrolyzed formulas, including PHWF from the same manufacturer as PHWF1 (33, 34), have been reported to retain the capacity to induce T-cell responses and oral tolerance (27). Although this study did not examine the ability of the two PHFs to activate T-cells, whey protein and casein hydrolysates contained in these PHFs can induce oral tolerance in skin sensitization models (35, 36). Furthermore, the efficacy of PHF2 in a clinical trial of oral immunotherapy for CMA suggests its potential in oral tolerance induction (30). Most children with CMA were sensitized to casein (18, 19). Since PHF1 has many casein IgE epitopes, it may have the potential to stochastically induce the activation of casein-specific T cells. As described above, CMA symptoms, including anaphylaxis, may develop with the first intake of cow's milk protein-containing meals, mainly CMF (14, 15). Therefore, as a perspective on contribution to clinical practice, the use of PHF such as PHF1 and PHF2 at the first introduction may not only reduce the allergic symptoms when ingested by infants with CMA before they are diagnosed with CMA but could also enable ingestion of cow's milk protein by inducing oral tolerance without developing any symptoms. It is unclear whether CMF or PHF has a greater preventive effect on CMA when the formula is administered continuously from the neonatal period. However, PHF could play an important role in the strategic prevention or treatment of CMA because of its ability to reduce CMA symptoms and induce oral tolerance. In the future, further research is required to confirm the safety of PHF1 and PHF2 by oral challenge tests, compare the ability of the PHFs to induce oral tolerance, and clarify the relationship between the allergenicity of casein and whey protein hydrolysates and the type of sensitizing allergen in patients with CMA.

## Data Availability

The original contributions presented in the study are included in the article/Supplementary Material, further inquiries can be directed to the corresponding author.
